# An Optimized Pathway for the Differential Diagnosis of ACTH-Dependent Cushing’s Syndrome Based on Low-Dose Dexamethasone Suppression Test

**DOI:** 10.3389/fendo.2021.720823

**Published:** 2021-09-02

**Authors:** Kang Chen, Shi Chen, Lin Lu, Huijuan Zhu, Xiaobo Zhang, Anli Tong, Hui Pan, Renzhi Wang, Zhaolin Lu

**Affiliations:** ^1^Department of Endocrinology, Key Laboratory of Endocrinology of National Health Commission, Translation Medicine Centre, Peking Union Medical College Hospital, Peking Union Medical College, Chinese Academy of Medical Sciences, Beijing, China; ^2^Eight-Year Program of Clinical Medicine, Peking Union Medical College Hospital, Peking Union Medical College, Chinese Academy of Medical Sciences, Beijing, China; ^3^Department of Radiology, Peking Union Medical College Hospital, Peking Union Medical College, Chinese Academy of Medical Sciences, Beijing, China; ^4^State Key Laboratory of Complex Severe and Rare Diseases, Peking Union Medical College Hospital, Chinese Academy of Medical Science and Peking Union Medical College, Beijing, China; ^5^Department of Neurosurgery, Peking Union Medical College Hospital, Peking Union Medical College, Chinese Academy of Medical Sciences, Beijing, China

**Keywords:** Cushing’s disease, ectopic ACTH syndrome, dexamethasone suppression test, petrosal sinus sampling, ROC curve

## Abstract

**Context:**

Traditionally, low-dose dexamethasone suppression test (LDDST) was used to confirm the diagnosis of Cushing’s syndrome (CS), and high-dose dexamethasone suppression test (HDDST) was used to differentiate Cushing’s disease (CD) and ectopic adrenocorticotropin (ACTH) syndrome (EAS), but some studies suggested that HDDST might be replaced by LDDST. For the differential diagnosis of CS, dexamethasone suppression test was usually combined with other tests such as bilateral petrosal sinus sampling (BIPSS) and pituitary magnetic resonance imaging, but the optimal pathway to incorporate these tests is still controversial.

**Objectives:**

To develop an optimized pathway for the differential diagnosis of CD and EAS based on LDDST.

**Design and Setting:**

Single-center retrospective study (2011–2019).

**Patients:**

Two hundred sixty-nine CD and 29 EAS patients with pathological diagnosis who underwent consecutive low- and high-dose DST.

**Results:**

For the differential diagnosis of CD and EAS, the area under curve (AUC) of LDDST using urine free cortisol (0.881) was higher than that using serum cortisol (0.685) (p < 0.001) in head-to-head comparison among a subgroup of 108 CD and 10 EAS. The AUC of LDDST (0.883) was higher than that of HDDST (0.834) among all the included patients. With the cutoff of <26%, the sensitivity and specificity of LDDST were 39.4% and 100%. We designed a new pathway in which BIPSS was only reserved for those patients with unsuppressed LDDST and adenoma <6mm, yielding an overall sensitivity of 97.7% and specificity of 86.7%.

**Conclusion:**

LDDST had similar value to HDDST in differentiating CD and EAS using the specific cutoff point. The pathway that combined LDDST and BIPSS could differentiate CD and EAS accurately.

## Introduction

Cushing’s disease (CD) and ectopic ACTH syndrome (EAS) are two main causes of ACTH-dependent Cushing’s syndrome (CS) (1). Their clinical manifestations are similar, but the treatments for them are quite different, so the differential diagnosis of CD and EAS is a crucial but challenging task.

Dexamethasone suppression test was first introduced by Liddle in 1960 (2). While normal hypothalamus–pituitary–adrenal axis was regulated by negative feedback, the cortisol secretion in CS was partly resistant to excess glucocorticoid, and it was believed that the ectopic tumor has higher autonomy of ACTH secretion compared with the pituitary tumor (1, 3). Based on these characteristics, low-dose dexamethasone suppression test (LDDST) was designed to diagnose CS and high-dose dexamethasone suppression test (HDDST) to differentiate CD and EAS (4). In standard HDDST, 24-h urine before and after a 2-day administration of dexamethasone (5) is time consuming and usually needs hospitalization. Modifications was made to simplify the procedure, such as the measurement of morning cortisol instead of 24-h urine free cortisol (UFC) or the overnight administration of 8-mg dexamethasone (4, 6). The sensitivity and specificity were highly variable among studies, both ranging from 60% to 100% (3, 4). Some sophisticated criteria to interpret the HDDST results was developed to improve its accuracy, but their reproducibility seemed to be poor (4, 7). Some studies found that HDDST provided little information for the differential diagnosis of CD and EAS and even suggested that it might be abandoned (8, 9). Moreover, the administration of high-dose dexamethasone in CS patients with already high cortisol level may lead to some side effects, exacerbating their hypertension and glucose metabolism disorder. Thus, the necessity of HDDST should be questioned. Meanwhile, it was proposed that LDDST per se may be an alternative for the differential diagnosis of ACTH-dependent Cushing’s syndrome (7, 10, 11), but such viewpoint has not been widely accepted.

Dexamethasone suppression test alone was not accurate enough to discriminate CD and EAS, so a diagnostic pathway that incorporate several diagnostic tests was necessary (3). In the commonly used pathway, corticotropin-releasing hormone (CRH) test, HDDST, pituitary MRI, and bilateral petrosal sinus sampling (BIPSS) were combined to establish the cause of ACTH-dependent CS (1, 12). It was reported that HDDST combined with CRH stimulation test could yield satisfactory accuracy, but CRH is not available in many districts, and the interpretation of CRH test was confusing (1, 12–14). BIPSS is another powerful diagnostic tool with high sensitivity and specificity, but its invasiveness and high cost limit its wide application, and the indication for BIPSS was still controversial (15–17). Besides, the traditional pathway was time consuming, mainly due to the HDDST.

The aim of the present study is to develop an optimized pathway for the differential diagnosis of CD and EAS based on the available tests. In this study, we analyze the data of consecutive low- and high-dose dexamethasone suppression test and BIPSS in a large series, and compared the accuracy of the traditional pathway and our new pathway.

## Materials and Methods

### Patients

Data were retrospectively collected from patients who were evaluated in Peking Union Medical College Hospital from 2011 to 2019. All of the included patients underwent consecutive low- and high-dose dexamethasone suppression test, and their final diagnosis of CD or EAS were pathologically confirmed after surgery or biopsy. The Institutional Review Board of Peking Union Medical College Hospital, Chinese Academy of Medical Sciences approved this study (approval number ZS-1083), and all the patients gave their informed consent for the use of their data.

For patients with suspected CS, serum cortisol, 24-h UFC, and LDDST were routinely conducted to confirm or exclude the diagnosis of CS. Patients were diagnosed as CS if the 24-h UFC after LDDST was not suppressed to below the lower limit of reference interval (12.3 μg). Experienced endocrinologists evaluate the history of the patients, and the onset of symptoms related to hypercortisolism was used to calculated the duration of the disease. After that, ACTH measurement, HDDST, pituitary dynamic enhanced MRI, and BIPSS were conducted as needed to establish the cause of CS. While overnight LDDST can be conducted for outpatients, “standard” 2-day LDDST was usually repeated after hospitalization, most of which were follow by HDDST immediately (see below) in our center. To avoid potential influence from the fluctuation of cortisol secretion, patients who underwent LDDST and HDDST separately were not included in this study.

### Dexamethasone Suppression Test

Consecutive low- and high-dose dexamethasone suppression test was conducted according to the protocol by Flack et al. (5). Twenty-four-hour urine on days 1 and 2 was collected, and their average UFC was the baseline. On days 3 and 4, 0.5 mg dexamethasone was administered every 6 h (low-dose), and 24-h UFC was measured on day 4. On days 5 and 6, 2 mg dexamethasone was administered every 6 h (high-dose), and 24-h UFC was measured on day 6. The ratio of 24-h UFC of day 4 and baseline was the result of LDDST, and the ratio of day 6 and baseline was the result of HDDST. In part of the patients, serum cortisol was measured in the morning of days 1, 2, 5, and 7, and the results of LDDST and HDDST were also calculated according to the serum cortisol. Their cutoff values are discussed in detail in the following text.

### MRI

Dynamic contrast-enhanced MRI of pituitary was conducted routinely. When distinct hypoenhanced lesion was detected (18), the MRI result was considered to be positive, and the maximum dimension of the lesion was recorded.

### BIPSS

The BIPSS procedures were all conducted by the same team of experienced radiologist according to the protocol described by Doppman et al. (19). In brief, catheters were guided into inferior petrosal sinus (IPS) through bilateral femoral veins, and blood samples were collected from peripheral vein and bilateral IPS simultaneously at baseline and 3, 5, and 10 min after desmopressin (10 μg iv) stimulation. There was no major complication among all the patients in this series. The IPS to peripheral ACTH ratio (IPS:P) was calculated. An IPS:P of more than 2 before stimulation or more than 3 at any time after stimulation supports the diagnosis of CD.

### Hormone Assay

Serum and urine cortisol was measured by direct chemiluminescence immunoassay (Siemens ADVIA Centaur). ACTH samples were delivered on ice and measured by chemiluminescence immunoassay (Siemens IMMULITE 2000).

### Comparison of Diagnostic Pathways

A subgroup were selected from the above-mentioned patients for the comparison of our new diagnostic pathway and the traditional pathway. The inclusion criteria were as follows: (i) the patient underwent BIPSS with desmopressin stimulation and (ii) the size of pituitary adenoma as measured by MRI were available. The accuracy of each pathway were retrospectively calculated according to these data. The traditional pathway was based on those reviewed by Lacroix et al. (1) and Sharma et al. (12), but the CRH stimulation test was omitted since it was unavailable in our center. In accordance with the recent guideline, BIPSS was not indicated among patients with tumor ≥6 mm on pituitary MRI in both the new and the traditional pathway in our study (16).

### Statistical Analysis

Normal variables decided by Shapiro–Wilk test were presented as average ± standard deviation and non-normal variables as median (first quartile, third quartile), and they were analyzed by t-test or non-parametric test, respectively. Chi-square test or Fisher’s exact test was used to analyzed categorical data. A p < 0.05 was considered statistically significant. Receiver operating characteristic (ROC) analysis was conducted, and the area under curve (AUC) was calculated to compare the diagnostic efficacy of different tests. For each diagnostic test, the cutoff to maximize the Youden index (sensitivity + specificity − 1) was calculated (20), and the cutoff to maximize the specificity. These analyses were performed using SPSS 25.0 and MedCalc 19.6.1.

## Results

### Characteristics of Patients

A total of 269 CD patients and 29 EAS patients were included in our study. The average age of all the included patients was 35.6 ± 12.7 years (range, 10–75 years). CD patients were predominantly female (84.4%), while 48.3% of the EAS patients were female (p < 0.001). Compared with the EAS patients, the CD patients had a longer duration of disease and lower morning cortisol, ACTH and 24-h UFC (all p < 0.001). Besides, the serum potassium level was significantly lower among the EAS patients (p = 0.003). Pituitary MRI results were positive among 88.4% (237/268) of the CD patients, while 25.0% (7/28) of the EAS patients also had positive MRI findings (p < 0.001). Their detailed clinical characteristics are presented in **Table 1**.

**Table 1:** Clinical characteristics of patients with CD and EAS.

	CD (n = 269)	EAS (n = 29)	p-value
Age (years)	35.6 ± 12.6	35.8 ± 13.8	0.951
Sex (male/female)	42:227 (0.19:1)	15:14 (1.07:1)	<0.001
BMI (kg/m^2^)	26.6 ± 4.2	25.7 ± 3.2	0.278
Duration of disease (months)	36 (24, 72)	12 (4, 25)	<0.001
Serum K+ (mmol/L)	3.8 ± 0.6	3.2 ± 0.8	0.003
Morning cortisol (μg/dl)	26.7 (21.7, 32.9)	35.4 (27.6, 52.0)	<0.001
ACTH (ng/L)	65.6 (45.9, 98.3)	135.0 (82.4, 238.0)	<0.001
24-h UFC (μg)	423.8 (279.0, 680.6)	1,280.9 (396.4, 2,299.8)	<0.001
Positive pituitary MRI	88.4% (237/268)	25.0% (7/28)	<0.001

For the 29 EAS patients, the ectopic tumor was mainly located at lung (15 patients, 51.7%) or mediastinum (10 patients, 34.5%). Three EAS cases were caused by pheochromocytoma, medullary thyroid carcinoma, and pelvic primitive neuroectodermal tumor, respectively (one patient for each type). The remaining one patient underwent biopsy of bone metastasis to establish the diagnosis of EAS, but the primary lesion was not pathologically confirmed.

### UFC and Serum Cortisol in DST

**Figure 1:**
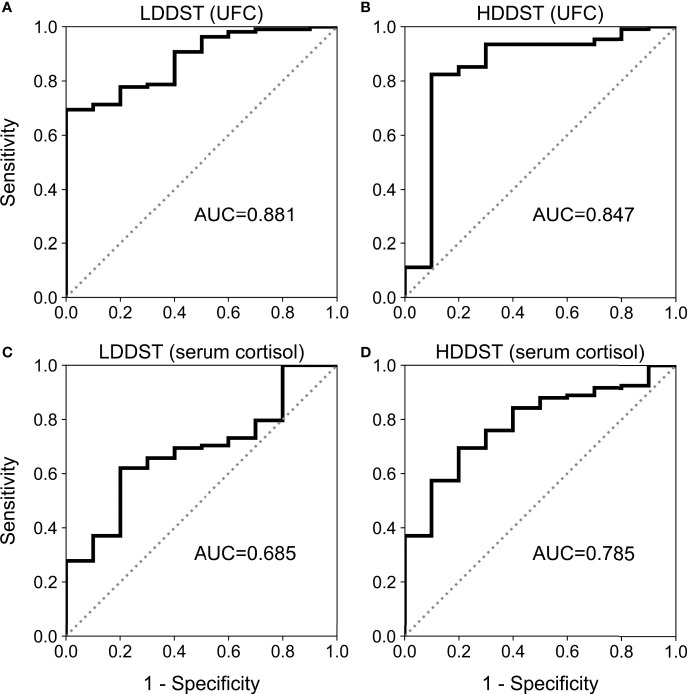
Receiver operating characteristic (ROC) curve for low-dose dexamethasone suppression test (LDDST) or high-dose dexamethasone suppression test (HDDST) using urine-free cortisol (UFC) **(A, B)** or serum cortisol **(C, D)** among 108 CD and 10 EAS patients with both serum and urine cortisol measurement during dexamethasone suppression test.

The data of both UFC and serum cortisol during DST were available among 108 CD patients and 10 EAS patients, and the diagnostic accuracy of LDDST and HDDST were compared among these patients (**Figure 1**). The LDDST calculated by UFC yielded an AUC of 0.881 (95%CI, 0.808–0.933), while that of LDDST by serum cortisol was 0.685 (95%CI, 0.593–0.768), the difference of which was significant (p < 0.001). The difference of HDDST by UFC or serum cortisol (0.847; 95%CI, 769–0.907 *vs*. 0.785, 95% CI, 0.700–0.855) was not significant (p = 0.210). Subsequent analyses on DST in this research were based on the results calculated by UFC.

### LDDST and HDDST

**Figure 2:**
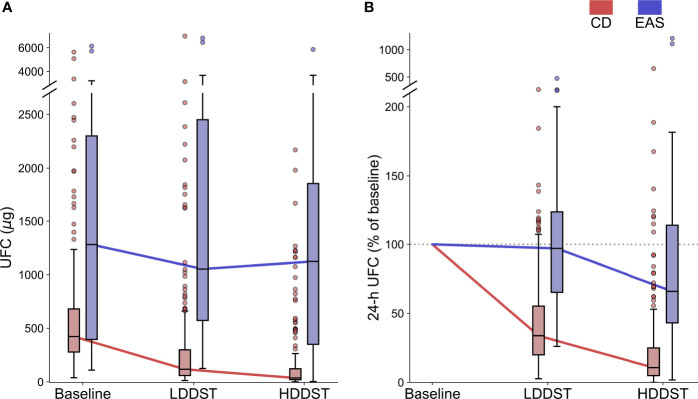
Change of UFC during consecutive low-dose dexamethasone suppression test (LDDST) and high-dose dexamethasone suppression test (HDDST) for CD (red) and EAS (blue) patients. **(A)** 24-h UFC at baseline, after LDDST, and after HDDST. **(B)** The ratio of 24-h UFC after dexamethasone suppression test and baseline. Box: interquartile range (IQR). Horizontal line inside each box: median. Whisker: maximum and minimum within median ± 1.5 × IQR. Circle: outlier outside 1.5 IQR.

After LDDST, the median 24-h UFC was 117.9 (59.2, 299.8) μg for CD and 1,053.4 (572.9, 2,450.0) μg for EAS (**Figure 2A**). The 24-h UFC were suppressed to a median of 33.9% (20.0%, 55.3%) of baseline among the CD patients and 97.0% (65.2%, 123.6%) of baseline among the EAS patients (**Figure 2B**). The greatest suppression among the EAS patients was 26.1% of baseline, while the greatest suppression among the CD patients was 2.7% of baseline.

After HDDST, the median 24-h UFC was 36.5 (20.4, 122.8) μg for the CD patients, and 1,123.7 (350.8, 1,856.4) μg for the EAS patients (**Figure 2A**). The 24-h UFC were suppressed to a median of 10.7% (4.9%, 25.0%) of baseline for the CD patients and 66.1% (43.2%, 114.0%) of baseline for the EAS patients (**Figure 2B**). Thirteen CD patients were suppressed to undetectable level, while the lowest 24-h UFC after HDDST among the EAS patients was 3.5 μg (1.8% of baseline).

### ROC Analysis and Diagnostic Accuracy

**Figure 3:**
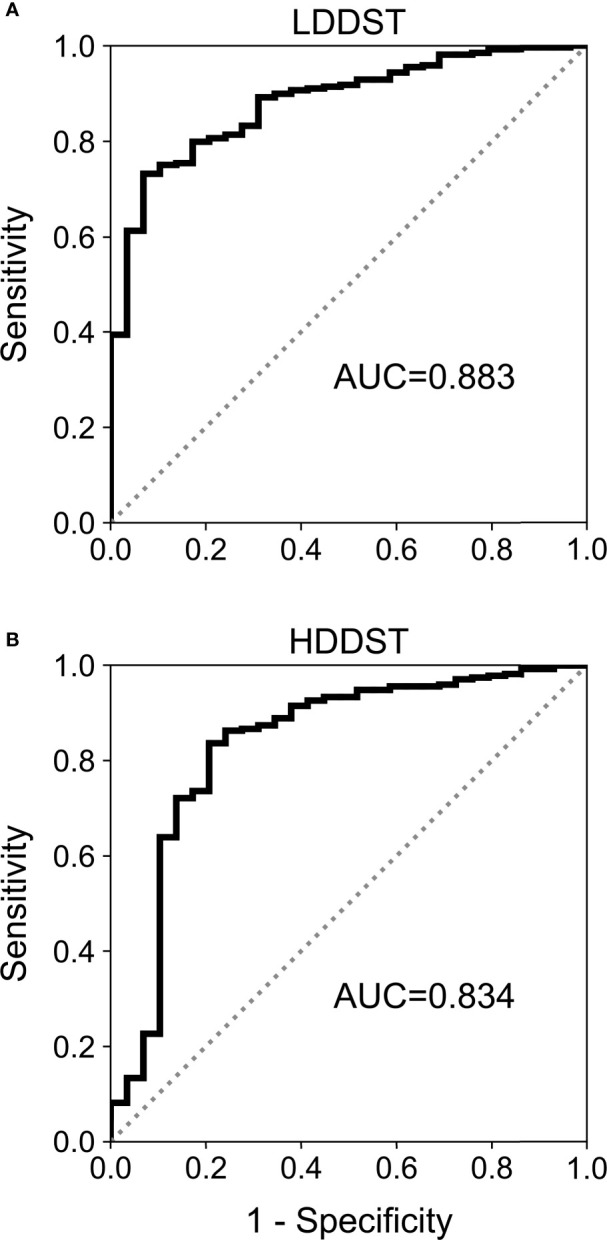
Receiver operating characteristic (ROC) curves for **(A)** low-dose dexamethasone suppression test (LDDST) and **(B)** high-dose dexamethasone suppression test (HDDST) among all the included patients.

The results of ROC analysis are demonstrated in **Figure 3**. The area under curve (AUC) for LDDST was 0.883 (95%CI, 0.840–0.916). The AUC for HDDST was 0.834 (95%CI, 0.787–0.874).

The cutoff to maximize the Youden index was suppressed to <52.3% of baseline for LDDST, and was <37.6% of baseline for HDDST. The cutoff to maximize the specificity was suppressed to <26.0% of baseline for LDDST and was <1.7% of baseline for HDDST. The corresponding values of sensitivity and specificity are listed in **Table 2**. While retaining 100% specificity, the highest sensitivity was reached when the cutoff of <26% of baseline after LDDST was used, which yield a sensitivity of 39.4%. On the contrary, the sensitivity of HDDST was only 7.8% when the cutoff to retain 100% specificity (<1.7% of baseline) was used. The commonly used cutoff for HDDST (<50% of baseline) yielded a sensitivity of 90.0% and a specificity of only 62.1%.

**Table 2:** Utility of low- and high-dose dexamethasone suppression test for the differential diagnosis of CD and EAS.

Criteria for suppression	CD, suppressed (TP)	EAS, not suppressed (TN)	EAS, suppressed (FP)	CD, not suppressed (FN)	Sensitivity % (95%CI)	Specificity % (95%CI)
LDDST	197	27	2	72	73.2	93.1
<52.3%					(67.5, 78.4)	(77.2, 99.2)
LDDST	106	29	0	163	39.4	100
<26.0%					(33.5, 45.5)	(88.1, 100)
HDDST	225	23	6	44	83.6	79.3
<37.6%					(78.7, 87.9)	(60.3, 92)
HDDST	242	18	11	27	90.0	62.1
<50%					(85.7, 93.3)	(42.3, 79.3)
HDDST	21	29	0	248	7.8	100
<1.7%					(4.9, 11.7)	(88.1, 100)

TP, true positive; TN, true negative; FP, false positive; FN, false negative.

### Comparison of CD Patients who Were Suppressed or Unsuppressed During LDDST

**Figure 4:**
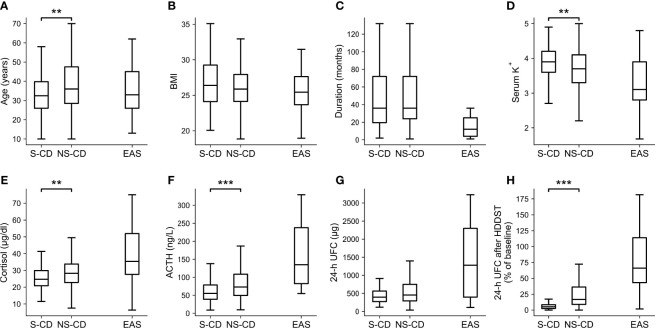
Clinical characteristics of the CD patients whose UFC was suppressed (S-CD) or cannot be suppressed (NS-CD) to <26% of baseline during LDDST and the EAS patients. **(A)** Age. **(B)** Body mass index (BMI). **(C)** Duration of disease. **(D)** Serum potassium level. **(E)** Morning cortisol. **(F)** ACTH. **(G)** 24-hour urine free cortisol (UFC). **(H)** 24-h UFC after high-dose dexamethasone suppression test. Box: interquartile range (IQR). Line inside the box: median. Whisker: maximum and minimum within median ± 1.5 × IQR. Outliers outside 1.5 IQR were not shown. Comparisons were only made between S-CD and US-CD but not EAS. *p < 0.05. **p < 0.01. ***p < 0.001.

When the cutoff of <26% of baseline was adopted during LDDST, 106 CD patients were suppressed while 163 CD patients were not suppressed, and the comparisons of their clinical characteristics along with EAS patients are shown in **Figure 4** (significance of difference was only calculated for suppressed and unsuppressed CD patients). The body mass index (BMI) (p = 0.257), duration of disease (p = 0.722), and 24-h UFC (p = 0.063) of suppressed and unsuppressed CD patients were not significant different. Among the CD patients who cannot be suppressed during LDDST, the serum potassium level (p = 0.006) were significantly lower. In contrast, their age (p = 0.001), morning cortisol (p = 0.008), ACTH level (p < 0.001), and 24-h UFC after HDDST (p < 0.001) were significantly higher than those who were suppressed during LDDST. Besides, 13.2% (14/106) of the suppressed patients were male, while 17.2% (28/163) of the unsuppressed patients were male (p = 0.397).

### Comparison of New and Traditional Diagnostic Pathway

A subgroup of 146 patients were selected for the comparison of the two diagnostic pathway, including 131 CD patients and 15 EAS patients. The age, BMI, duration of disease, morning cortisol, ACTH, and 24-h UFC were not significantly different between the selected and the excluded patients (data not shown).

**Figure 5:**
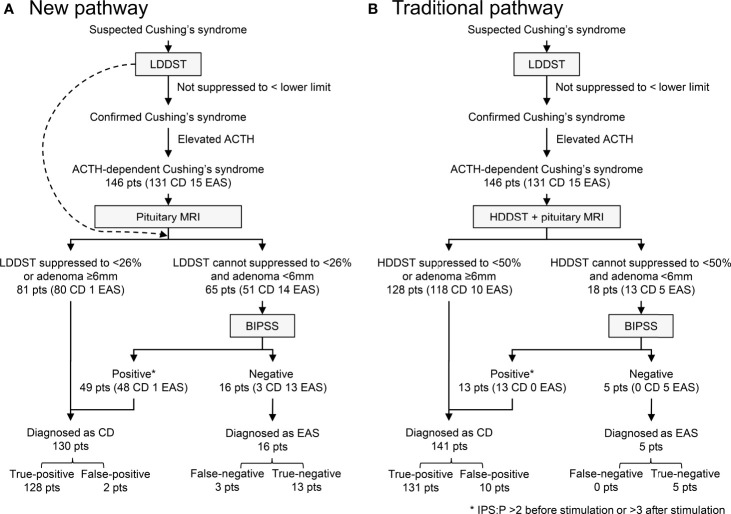
Diagnostic utility of new **(A)** and traditional **(B)** pathway. IPS:P refers to inferior petrosal sinus to peripheral ACTH gradient.

Our new diagnostic pathway that incorporated LDDST and BIPSS was illustrated in **Figure 5A**. The overall sensitivity for this pathway was 97.7% (128/131), which was comparable to the sensitivity of the traditional pathway (100%, 131/131) (**Figure 5B**). The specificity of the new pathway was 86.7% (13/15), which was much higher than that of the traditional pathway (33.3%, 5/15). In the new pathway, BIPSS was needed in 65 (44.5%) patients, while 18 (12.3%) patients needed BIPSS in the traditional pathway.

## Discussion

To the best of our knowledge, this is the largest series to compare the value of LDDST and HDDST in discriminating CD and EAS among pathologically confirmed cases. We found that LDDST could not only differentiate CD and EAS with higher efficacy than HDDST but also reached a relatively high sensitivity when the specificity retained 100%. Based on this feature, a simplified pathway that incorporated LDDST could be designed for the differential diagnosis of ACTH-dependent CS, and HDDST might be abandoned to avoid potential side effects.

Before further discussion about LDDST and HDDST, the measures in these tests, that is, UFC or serum cortisol, should be clarified first. Although it was recommended to use serum cortisol in LDDST in diagnosis of CS (21), our study found that UFC performed better in LDDST for the differential diagnosis of CD and EAS. As for HDDST, our head-to-head comparison showed that serum cortisol and UFC had similar efficacy, which was consistent with previous studies that different measures were used in different patients or centers (22, 23). As a result, UFC was used in LDDST in this study and also in HDDST to ensure comparability.

The prevalence of CD is much higher than EAS (1), and conducting pituitary surgery in EAS patients due to misdiagnosis is more unacceptable than delaying the diagnosis of CD. Thus, an ideal test to distinguish CD from EAS should have a high specificity even with a compromised sensitivity. In this sense, HDDST might not be competent due to its unsatisfactory specificity. The difference in response to dexamethasone in CD and EAS was in a quantitative rather than a qualitative manner, and the cortisol secretion in some EAS patients can actually be suppressed to a very low level after HDDST in our study, which greatly reduced its specificity. Similarly, previous studies also reported that HDDST could hardly reach a high specificity with an acceptable sensitivity (3, 4, 9). On the contrary, EAS patients only showed minimal suppression during LDDST, while quite a few CD patients was greatly suppressed. In fact, LDDST not only had higher AUC but also better specificity with enough sensitivity in our study when an appropriate cutoff was selected, which was in concordance with the study by Isidori et al. (7). With a high specificity, patients with suppressed LDDST could be diagnosed as CD almost without exception.

After LDDST with our high-specificity cutoff, only a part of the CD patients along with all the EAS patients needed further tests to establish their causes of CS, but this is a more difficult task since this subgroup of patients were quite similar. According to our observations, the pituitary tumors in these CD patients with unsuppressed LDDST might be more active and autonomous than those that can be suppressed. They produced more ACTH and responded less to the negative feedback from glucocorticoid, mimicking the behavior of ectopic tumors to some extent. Thus, conducting HDDST after LDDST could hardly produce additional information for the differential diagnosis.

Based on the combination of LDDST, pituitary MRI, and BIPSS, we designed a new pathway for the differential diagnosis of CD and EAS. After establishing the diagnosis of ACTH-dependent CS, LDDST should be the first test to schedule. Patients whose 24-h UFC can be suppressed to <26% of baseline during LDDST are considered as “typical” CD, and further diagnostic tests are unnecessary for them. Otherwise, patients with unsuppressed LDDST should consider BIPSS. Further selection was made according to the recent guideline (16), so BIPSS was only indicated for patients with unsuppressed LDDST and tumor below 6 mm. If BIPSS is not available or the patients refuse such invasive test, LDDST can also be a substitute for HDDST since LDDST has higher AUC. In this scenario, the cutoff with maximal Youden index can be adopted to balance the sensitivity and the specificity.

Compared with the traditional pathway, this new pathway could discriminate CD and EAS with a similar sensitivity but much higher specificity. The time to establish the diagnosis was much shorter, and most of the tests could even be finished in outpatient. Moreover, the side effect of glucocorticoid such as fluctuation in blood pressure or blood glucose could be minimized, since only a small dose of dexamethasone was administered during LDDST.

The combination of multiple tests is necessary for the accurate and robust differential diagnosis of CD and EAS. The most valuable tests include biochemical test like DST and CRH stimulation test, imaging studies such as pituitary dynamic enhanced MRI and radioisotope studies, and BIPSS (24). BIPSS was a reliable test for the differential diagnosis of CD and EAS with an excellent sensitivity and a specificity of near 100% (17, 25). However, BIPSS is invasive and expensive, and currently, it is not widely available. Thus, identifying those who need BIPSS most is a critical step in the diagnostic pathway. A well-established strategy is to waive BIPSS among those patients with adenoma over 6 mm on MRI plus concordant HDDST and CRH stimulation test (1, 12). However, CRH test is also not widely available, and HDDST is time consuming and complicated. Luckily, the current study found that LDDST in combination with pituitary MRI might serve as a filter to ruled out those “typical” CD who could be correctly diagnosed without BIPSS, which was more accurate and convenient than the traditional pathway based on the combination of HDDST and MRI in the absence of CRH test.

It should be noticed that the new pathway in this study may not be the optimal one. On the one hand, a pituitary adenoma over 6 mm was observed in some EAS patients in the current series, so this cutoff may need optimization. On the other hand, it was regrettable that CRH test was not included in our pathway. CRH test is more convenient than BIPSS, and the combination of CRH test and HDDST was reported to be highly accurate (12–14). However, CRH stimulation test was not carried out since CRH was not available in our area. We believe that the incorporation of CRH test in our pathway might further improve the accuracy and reduce the reliance on BIPSS. The combination of LDDST and CRH test can be validated in centers where CRH is available.

Our study had some limitations. Some patients, especially EAS patients, underwent LDDST and HDDST separately in our center. These patients were not included in this study, and it is unclear whether they had major difference with the included patients. The comparison of pathways was also conducted retrospectively, and some “typical” patients did not undergo BIPSS. These factors might introduce selection bias. Besides, this is a single-center study, and the cutoff should be validated in other centers.

In conclusion, the optimized pathway that combined LDDST, pituitary MRI, and BIPSS could differentiate CD and EAS accurately. LDDST could effectively identify the cases who were difficult to differentiate and really needed advanced tests such as BIPSS, and thus, it might replace HDDST to save several days of examination and to prevent risks due to elevated cortisol, making the pathway simpler.

## Data Availability Statement

The raw data supporting the conclusions of this article will be made available by the authors, without undue reservation.

## Ethics Statement

The studies involving human participants were reviewed and approved by Institutional Review Board of Peking Union Medical College Hospital, Chinese Academy of Medical Sciences. Written informed consent to participate in this study was provided by the participants’ legal guardian/next of kin.

## Author Contributions

KC collected and analyzed the data and prepared the manuscript. SC and LL conceptualized the study and revised the manuscript. HZ, XZ, AT, and RW managed the patients and revised the manuscript. HP and ZL supervised the study. All authors contributed to the article and approved the submitted version.

## Funding

This work was supported by the CAMS Innovation Fund for Medical Science (grant number CAMS-2017-I2M-1-011) and the National Key Research and Development Program of China (grant number 2016YFC0901500).

## Conflict of Interest

The authors declare that the research was conducted in the absence of any commercial or financial relationships that could be construed as a potential conflict of interest.

## Publisher’s Note

All claims expressed in this article are solely those of the authors and do not necessarily represent those of their affiliated organizations, or those of the publisher, the editors and the reviewers. Any product that may be evaluated in this article, or claim that may be made by its manufacturer, is not guaranteed or endorsed by the publisher.
